# Mediation by Lipid‐Glucose Metabolic Indices in the Association Between Overweight and T2DM Among Shanghai Community‐Dwelling Older Adults

**DOI:** 10.1155/jdr/8449810

**Published:** 2026-06-26

**Authors:** Jia Bo, Hongfei Mo, Fei Wu, Jiayun Zhu, Qinghua Yan, Fan Wang, Minna Cheng

**Affiliations:** ^1^ School of Nursing and Health Management, Shanghai University of Medicine & Health Sciences, Shanghai, China, sumhs.edu.cn; ^2^ School of Public Health, Fudan University, Shanghai, China, fudan.edu.cn; ^3^ Fudan Development Institute, Fudan University, Shanghai, China, fudan.edu.cn; ^4^ Division of Chronic Non-Communicable Disease and Injury, Shanghai Municipal Center for Disease Control and Prevention, Shanghai, China, scdc.sh.cn

**Keywords:** lipid-glucose metabolic indices, mediation analysis, older adults, overweight, Type 2 diabetes mellitus (T2DM)

## Abstract

**Objective:**

This study is aimed at exploring the mediating roles of the triglyceride‐glucose (TyG) index, cholesterol‐HDL‐glucose (CHG) index, and atherogenic index of plasma (AIP) in the association between overweight and Type 2 diabetes mellitus (T2DM) among older adults in Shanghai, China.

**Methods:**

A total of 44,715 participants aged ≥ 60 years from the Shanghai Non‐communicable Diseases Management Cohort were included. Overweight was defined as a body mass index (BMI) ≥ 24 kg/m^2^. T2DM was diagnosed clinically by physicians. Mediation analysis was performed to estimate direct, indirect, and total effects.

**Results:**

The mean age of the participants was 77 ± 6.8 years, 16,835 (37.65%) were diagnosed with T2DM, and 27,880 (62.35%) did not. In covariate‐adjusted models, overweight older adults had a 26% higher odds of T2DM than nonoverweight older adults. The TyG, CHG, and AIP indices were all associated with T2DM. In the mediation analysis, the proportions of the total effect mediated by the TyG, CHG, and AIP indices were 22.03%, 32.24%, and 6.47%, respectively.

**Conclusion:**

The lipid‐glucose metabolic indices examined, particularly CHG and TyG indices, partially mediated the association between overweight and T2DM. These biomarkers may serve as early intervention targets to prevent T2DM among overweight older adults.

## 1. Background

Type 2 diabetes mellitus (T2DM) is a major global public health issue. Its incidence continues to rise, and related conditions and mortality remain high. Previous studies reported that in 2021, there were 537 million adults living with diabetes worldwide, and this number is projected to reach 643 million by 2030, with T2DM accounting for about 90%~95% of these cases [1, 2]. T2DM is an important risk factor of cardiovascular diseases, chronic kidney disease, neuropathy, and retinopathy. These complications, together with disease‐related symptoms and treatment burden, greatly reduce quality of life and increase the risk of disabilities [3–5]. Global health spending on T2DM had reached 966 billion US dollars in 2021, and is expected to reach 1 trillion by 2030 [1].

The disease burden is especially heavy on older adults. Advancing age will lead to declining *β*‐cell function and rising insulin resistance, which expands the population at risk. This challenge is particularly severe in China, which has the largest number of people with diabetes in the world [6]. At the same time, the country is experiencing rapid population aging and increasing obesity rates, driven by urbanization, unhealthy diets, and physical inactivity [7]. The overall prevalence of T2DM in Chinese adults is estimated at 11.2%, representing over 114 million individuals. Among those aged 60 years and older, the prevalence has already exceeded 30% in some regions [8].

Excess body weight is a traditional risk factor for T2DM. Overweight (body mass index [BMI] ≥ 24 kg/m^2^) and obesity (BMI ≥ 30 kg/m^2^) are important causes of insulin resistance, which act as a central defect behind T2DM [3]. In addition, adipose tissue, especially visceral fat, releases proinflammatory cytokines (such as IL‐6 and TNF‐*α*, nonesterified fatty acids, etc.), these molecules could disrupt insulin signaling in the liver and muscle, damage pancreatic *β*‐cell function, and promote hyperglycemia [1]. It has been estimated that obesity accounts for 80%~85% of the attributable risk for T2DM, and each 1 kg/m^2^ rise in BMI increases the risk by 12% [9]. However, not all individuals with obesity develop T2DM. This highlights the importance of how fat tissue functions and interacts with the body. The precise metabolic pathways linking excess body weight to T2DM are still not fully understood.

To capture these intermediate metabolic disturbances, previous studies have proposed composite lipid‐glucose indices. These include the triglyceride‐glucose (TyG) index, the cholesterol‐HDL‐glucose (CHG) index, and the atherogenic index of plasma (AIP). The TyG index is a validated marker of insulin resistance. Its estimation performance is comparable to that of the reference standard, the hyperinsulinemic‐euglycemic clamp [3, 10]. The CHG index gives a broader picture of combined dyslipidemia and hyperglycemia [11]. The AIP reflects a type of dyslipidemia that promotes atherosclerosis. It is characterized by high triglycerides and low high‐density lipoprotein cholesterol (HDL‐C), which is closely linked to insulin resistance and cardiovascular risk [12]. These indices may mediate the pathway from obesity to T2DM, as excess body weight induces insulin resistance and atherogenic dyslipidemia, which in turn contribute to the development of T2DM [13].

Previous studies have confirmed an association between a higher TyG index and the presence of T2DM [14, 15]. Similar associations have been reported for AIP and CHG [14, 16]. This suggests that these indices may reflect key metabolic disturbances that precede and contribute to the development of T2DM. However, it remains unclear how strong these associations are in community‐dwelling older adults and what patterns they show. Moreover, the mediating role of these indices in the overweight‐T2DM pathway has not been quantified in this population. Evaluating and comparing all three indices together in a single study can help identify the most informative biomarker and offer mechanistic insight.

Therefore, this study is aimed at: (1) evaluating the independent associations of TyG index, CHG index, and AIP with T2DM among older adults in Shanghai; and (2) quantifing the extent to which these indices mediate the association between overweight and T2DM. The findings from this study are intended to identify potential metabolic links between excess body weight and T2DM in older adults. They may also inform future risk assessment and screening approaches that use these simple indices in populations with a high prevalence of T2DM.

## 2. Materials and Methods

### 2.1. Data Source and Study Population

This study utilized data from the Shanghai Non‐communicable Diseases (NCDs) Management Cohort, a large‐scale, community‐based survey that is continuously maintained by the Shanghai Municipal Center for Disease Control and Prevention (SCDC). The digitized chronic disease management system supports standardized care and big data analysis for major NCDs, including hypertension, diabetes, dyslipidemia, and so on. It thus informs public health policy and research across Shanghai [17]. Data collection has been ongoing since 2020. For this analysis, we included participants enrolled between January 1, 2020, and December 31, 2024. All variables were assessed at a single time point using data extracted directly from existing electronic records. No supporting information collection, secondary record review, or follow‐up was conducted.

The parent database contained 3,505,657 individuals registered during this period. From this source population, 50,000 participants were selected by simple random sampling without replacement. We used the “sample” function in R with a fixed seed to ensure reproducibility. This sample size provided adequate statistical power for the planned mediation analyses while remaining computationally feasible for rigorous data cleaning and modeling. The randomly drawn subset formed the initial analytical sample.

**Figure 1:**
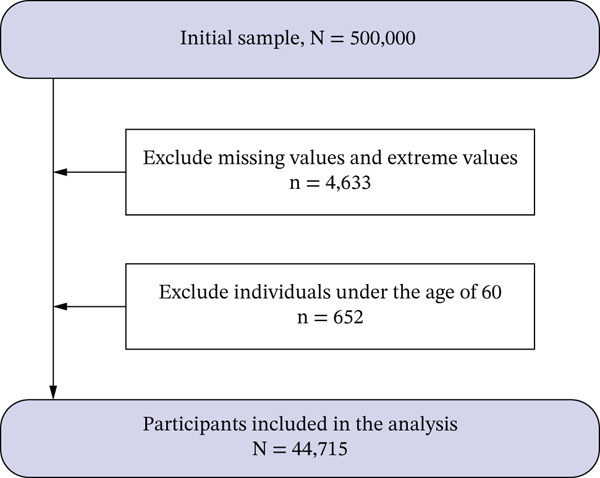
Sample selection flow chart.

Participants were excluded based on the following criteria: (1) missing or incomplete data on key variables (BMI, fasting blood glucose, lipid profile, or demographic characteristics); (2) extreme, physiologically implausible values (BMI < 15 or > 50 kg/m^2^, fasting glucose > 300 mg/dL). A total of 4633 participants were excluded for these reasons. In addition, 652 individuals aged < 60 years were excluded to focus the study on the older adult population. After all exclusions, the final analytical sample consisted of 44,715 community‐dwelling older adults (Figure 1).

The study was conducted in accordance with the Declaration of Helsinki. Ethics approval was obtained from the Ethics Committee of SCDC (approval number: KY‐2024‐52‐C). All participants provided written informed consent before participation.

### 2.2. Dependent Variable Assessment

Diagnosis of T2DM was based on the International Classification of Diseases, 11th Revision (ICD‐11) criteria [7] and the American Diabetes Association guidelines [18]. T2DM was defined by any of the following: (1) fasting plasma glucose ≥ 7.0 mmol/L; (2) 2‐hour plasma glucose during an oral glucose tolerance test ≥ 11.1 mmol/L; (3) glycated hemoglobin ≥ 6.5%; (4) random plasma glucose ≥ 11.1 mmol/L accompanied by classic symptoms of hyperglycemia. All diagnoses were physician‐confirmed and recorded using ICD‐11 codes in the electronic medical record system. All cases were identified from the Shanghai NCDs Management Cohort, which integrates medical records across the city [2, 19]. Verified health system records were used instead of self‐reported or single‐test diagnoses, in order to reduce misclassification and enhance the reliability of T2DM ascertainment [1].

### 2.3. Independent Variable Assessment

The primary independent variable was overweight status (overweight vs. nonoverweight), defined by BMI. Body measurements were performed by trained staff at community health examination centers, following the standardized protocols of the Shanghai NCDs Management Cohort. Height was recorded to the nearest 0.1 cm, and weight to the nearest 0.1 kg [20]. According to the criteria of the Working Group on Obesity in China, overweight was defined as BMI ≥ 24 kg/m^2^ [21]. This cutoff is widely used for Chinese adults and is associated with increased cardiometabolic risk [4, 22]. Participants with a BMI < 24 kg/m^2^ were classified as nonoverweight.

### 2.4. Mediating Variable Assessments

The mediating variables included the TyG index, CHG index, and AIP. These composite indices are derived from routine blood lipid and glucose measurements. After a 10‐hour overnight fast, venous blood was collected from the antecubital vein by trained healthcare staff. All samples were transported to the Central Laboratory of SCDC within 2 h. Fasting plasma glucose, triglycerides, total cholesterol (TC), and HDL‐C were measured using enzymatic methods on an automated biochemistry analyzer. For the calculation of the composite indices, all lipid and glucose values were expressed in mg/dL. Low‐density lipoprotein cholesterol (LDL‐C) was estimated using the Friedewald formula: LDL − C (mg/dL) = TC − HDL − C − TG/5 [23]. Quality control was implemented throughout to ensure accuracy and comparability.

The three indices were calculated using the following formulas [24, 25]:

1. TyG index = Ln (fasting triglycerides (mg/dL) × fasting glucose (mg/dL)/2)
2. CHG index = Ln (TC (mg/dL) × fasting glucose (mg/dL)/(2 × HDL‐C (mg/dL))
3. AIP = Log (triglycerides (mg/dL)/HDL‐C (mg/dL))

### 2.5. Covariates

Covariates were selected a priori based on their known associations with overweight, lipid‐glucose metabolism, and T2DM. To avoid overadjustment for potential mediators, we included only basic demographic and life behavioral variables in the primary analysis. Age was modeled as a continuous variable. All categorical covariates were entered as dummy variables, including gender (male, female), marital status (married, not married), education level (primary school or below, junior high school, high school or above), smoking status (*never*, *former*, *current smoker*), alcohol consumption (*no*, *yes*), and physical activity (*no*, *yes*).

### 2.6. Statistical Analyses

Statistical analyses were performed to investigate the associations between overweight, lipid‐glucose metabolic indices, and T2DM. Participants were divided into T2DM and nonT2DM groups.

All continuous variables were assessed for normality using the Shapiro–Wilk test and were found to follow a normal distribution. They are therefore presented as mean ± standard deviation (SD) and compared with two‐sample *t*‐tests. Categorical variables are summarized as frequencies (percentages) and compared using chi‐square tests.

Logistic regression was used to assess the associations of overweight and each mediator index (TyG, CHG, AIP) with T2DM, and linear regression was used to examine the associations between overweight and each mediator index. All regressions were fitted in two specifications: a crude model (Model I) and a covariates‐adjusted model (Model II) that included age, sex, marital status, education, smoking, alcohol consumption, and physical activity. Restricted cubic splines (RCS) with knots placed at the 5th, 35th, 65th, and 95th percentiles were used to characterize dose‐response relationships and were implemented using the “rms” package in R.

Mediation analysis was conducted under the potential outcomes framework using the “mediation” package [26]. For each mediator, the total effect (TE) of overweight on T2DM was decomposed into the average causal mediation effect (ACME, i.e., indirect effect) and the average direct effect (ADE). The proportion mediated (PM) was calculated as ACME/TE. Quasi‐Bayesian Monte Carlo simulation with 1000 draws was used to obtain point estimates and bias‐corrected confidence intervals for all effects.

Model assumptions were examined as follows. For linear regression, residual normality and homoscedasticity were checked with Q‐Q plots and the Breusch–Pagan test. Variance inflation factors were inspected to rule out multicollinearity. For logistic regression, goodness of fit was assessed with the Hosmer–Lemeshow test, and discrimination was evaluated by the area under the receiver operating characteristic curve (AUC).

All analyses were performed using R software Version 4.3.0. A two‐tailed significance level of *α* = 0.05 was applied.

## 3. Results

### 3.1. Demographic Characteristics of Participants

A total of 44,715 participants were included in the final analysis, 16,835 (37.65%) were diagnosed with T2DM, and 27,880 (62.35%) did not. Among the participants, 20,853 (46.64%) were male and 23,862 (53.36%) were female. The overall mean age was 77.20 ± 6.82 years.

Participants with T2DM had a significantly higher BMI than those without T2DM (24.22 ± 2.93 vs. 23.85 ± 2.86 kg/m^2^). They also had a higher rate of overweight (49.89% vs. 44.09%), the differences were statistically significant (*p* < 0.001). Regarding glycolipid metabolic indices, the T2DM group showed significantly higher levels of fasting glucose, triglycerides, TyG index, CHG index, and AIP, as well as significantly lower HDL‐C (all *p* < 0.001).

Among the covariates, several showed significant differences between the T2DM and nonT2DM groups, including sex, marital status, education level, alcohol consumption, and physical activity (*p* < 0.001). No significant differences were found for age or smoking status. The detailed demographic characteristics of the study population, stratified by T2DM status, are presented in Table 1.

**Table 1:** Demographic characteristics of participants, by T2DM.

Variables	Sample *n* = 44,715	T2DM		Statistic	*p*
		No, *n* = 27,880	Yes, *n* = 16,835		
Age (years), mean ± SD	77.20 ± 6.82	77.17 ± 6.89	77.25 ± 6.69	*t* = −1.18	0.239
Sex, *n* (%)				*χ* ^2^ = 4.06	0.044
Male	20,853 (46.64)	12,899 (46.27)	7954 (47.25)		
Female	23,862 (53.36)	14,981 (53.73)	8881 (52.75)		
Marital, *n* (%)				*χ* ^2^ = 90.41	< 0.001
Married	35,673 (79.78)	21,851 (78.38)	13,822 (82.10)		
Not married	9042 (20.22)	6029 (21.62)	3013 (17.90)		
Education, *n* (%)				*χ* ^2^ = 25.81	< 0.001
Primary school and below	25,844 (57.80)	16,036 (57.52)	9,808 (58.26)		
Junior high school	8317 (18.60)	5060 (18.15)	3257 (19.35)		
High school and above	10,554 (23.60)	6784 (24.33)	3770 (22.39)		
Smoking, *n* (%)				*χ* ^2^ = 1.90	0.387
Never smoked	39,399 (88.11)	24,588 (88.19)	14,811 (87.98)		
Former smoker	2204 (4.93)	1344 (4.82)	860 (5.11)		
Current smoker	3112 (6.96)	1948 (6.99)	1164 (6.91)		
Alcohol, *n* (%)				*χ* ^2^ = 4.20	0.040
No	7271 (16.26)	4611 (16.54)	2660 (15.80)		
Yes	37,444 (83.74)	23,269 (83.46)	14,175 (84.20)		
Physical activity, *n* (%)				*χ* ^2^ = 196.70	< 0.001
No	33,052 (73.92)	21,239 (76.18)	11,813 (70.17)		
Yes	11,663 (26.08)	6,641 (23.82)	5,022 (29.83)		
BMI (kg/m^2^), mean ± SD	23.99 ± 2.89	23.85 ± 2.86	24.22 ± 2.93	*t* = −13.08	< 0.001
Overweight (BMI ≥ 24), *n* (%)				*χ* ^2^ = 142.09	< 0.001
No	24,024 (53.73)	15,588 (55.91)	8436 (50.11)		
Yes	20,691 (46.27)	12,292 (44.09)	8399 (49.89)		
Glucose (mg/dL), mean ± SD	111.73 ± 32.92	102.34 ± 21.62	127.29 ± 41.44	*t* = −72.38	< 0.001
TC (mg/dL), mean ± SD	188.03 ± 47.35	189.52 ± 46.85	185.57 ± 48.08	*t* = 8.49	< 0.001
TG (mg/dL), mean ± SD	164.00 ± 129.92	161.60 ± 128.58	167.97 ± 132.01	*t* = −4.99	< 0.001
HDL‐C (mg/dL), mean ± SD	52.27 ± 14.04	52.87 ± 14.05	51.28 ± 13.95	*t* = 11.64	< 0.001
LDL‐C (mg/dL), mean ± SD	109.56 ± 36.15	110.60 ± 35.79	107.84 ± 36.69	*t* = 7.79	< 0.001
TyG, mean ± SD	8.90 ± 0.65	8.81 ± 0.62	9.04 ± 0.68	*t* = −35.24	< 0.001
CHG, mean ± SD	5.26 ± 0.45	5.18 ± 0.41	5.40 ± 0.47	*t* = −32.90	< 0.001
AIP, mean ± SD	0.99 ± 0.69	0.96 ± 0.68	1.03 ± 0.69	*t* = −9.95	< 0.001

*Note:* Abbreviations are the same as in Table 2, Table 3, Table S1, Figure 2, Figure 3, and Figure S1.

Abbreviations: AIP, atherogenic index of plasma; BMI, body mass index; CHG, cholesterol‐HDL‐glucose index; HDL‐C, high‐density lipoprotein cholesterol; LDL‐C, low density lipoprotein cholesterol; SD, standard deviation; *t*, *t*‐test; T2DM, Type 2 diabetes mellitus; TC, total cholesterol; TG, triglyceride; TyG, triglyceride‐glucose index; *χ* ^2^, chi‐square.

### 3.2. Associations Between Overweight and Lipid‐Glucose Metabolic Indices

Linear regression was used to examine the associations between overweight and each lipid‐glucose metabolic index. In Model I, overweight was positively associated with the TyG index (*β* = 0.09, 95% CI: 0.08~0.11), the CHG index (*β* = 0.08, 95% CI: 0.06~0.09), and AIP (*β* = 0.11, 95% CI: 0.10~0.13). After adjustment for all covariates (Model II), these associations remained statistically significant: TyG index (*β* = 0.10, 95% CI: 0.09~0.11), CHG index (*β* = 0.07, 95% CI: 0.06~0.08), and AIP (*β* = 0.11, 95% CI: 0.10~0.13) (all *p* < 0.001) (Table 2).

**Table 2:** Linear association between overweight and lipid‐glucose metabolic indices.

Outcomes	Model I					Model II				
	*β*	S.E	*t*	*p*	*β* (95% CI)	*β*	S.E	*t*	*p*	*β* (95% CI)
TyG	0.09	0.01	15.21	< 0.001	0.09 (0.08~0.11)	0.10	0.01	15.84	< 0.001	0.10 (0.09~0.11)
CHG	0.08	0.01	12.12	< 0.001	0.08 (0.06~0.09)	0.07	0.01	11.38	< 0.001	0.07 (0.06~0.08)
AIP	0.11	0.01	17.56	< 0.001	0.11 (0.10~0.13)	0.11	0.01	17.34	< 0.001	0.11 (0.10~0.13)

*Note:* Model I, crude; Model II, adjusting for covariates.

Abbreviation: CI, confidence interval.

### 3.3. Associations Between Overweight and Lipid‐Glucose Metabolic Indices With T2DM

Logistic regression was used to evaluate the associations of overweight and the lipid‐glucose metabolic indices with T2DM. Overweight was significantly associated with higher odds of T2DM in both crude and adjusted models. In Model II, overweight individuals had 26% higher odds of T2DM (OR = 1.26, 95% CI: 1.21~1.31).

All three metabolic indices also showed significant positive associations with T2DM after full adjustment. Per unit increase, the TyG index was associated with a 72% higher odds of T2DM (OR = 1.72, 95% CI: 1.67~1.77), the CHG index with a 249% higher odds (OR = 3.49, 95% CI: 3.24~3.77), and the AIP with a 16% higher odds (OR = 1.16, 95% CI: 1.13~1.19) (Table 3).

**Table 3:** Logistic association between overweight and lipid‐glucose metabolic indices with T2DM.

Predictors	Model I					Model II				
	*β*	S.E	Z	*p*	OR (95% CI)	*β*	S.E	Z	*p*	OR (95% CI)
Overweight	0.23	0.02	11.91	< 0.001	1.26 (1.22~1.31)	0.23	0.02	11.70	< 0.001	1.26 (1.21~1.31)
TyG	0.53	0.02	34.88	< 0.001	1.71 (1.66~1.76)	0.54	0.02	35.11	< 0.001	1.72 (1.67~1.77)
CHG	1.24	0.04	32.53	< 0.001	3.46 (3.21~3.73)	1.25	0.04	32.32	< 0.001	3.49 (3.24~3.77)
AIP	0.14	0.01	9.97	< 0.001	1.15 (1.12~1.18)	0.15	0.01	10.25	< 0.001	1.16 (1.13~1.19)

*Note:* Model I, crude; Model II, adjusting for covariates.

Abbreviation: OR, odds ratio.

### 3.4. Dose–Response Relationships

**Figure 2:**
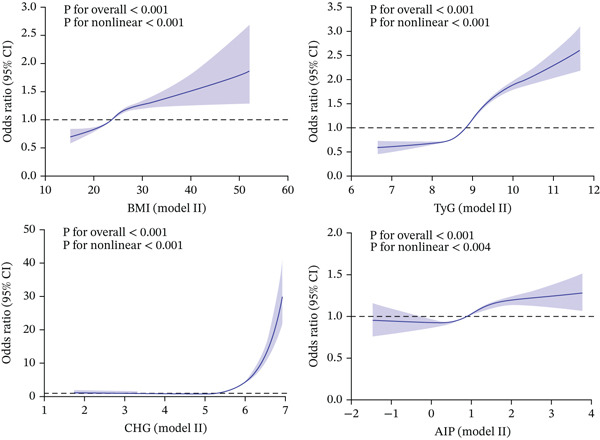
RCS curves of logistic association between BMI, lipid‐glucose metabolic indices, and T2DM in covariates‐adjusted models.

RCS were used to visualize the dose‐response relationships of BMI and the three lipid‐glucose metabolic indices (TyG, CHG, and AIP) with the log‐odds of T2DM after covariate adjustment. RCS analysis showed significant overall associations for all four variables (all *p* for overall < 0.001). The relationships of the TyG, CHG, and AIP indices with T2DM were all significantly nonlinear (all *p* for nonlinearity < 0.05), indicating that the magnitude of the increase in T2DM risk per unit change varied across the distribution of each index. Despite the nonlinearity, the fitted curves were monotonically positive, confirming that these indices act as continuous risk factors for T2DM (Figure 2).

### 3.5. Mediation by Lipid‐Glucose Metabolic Indices

**Figure 3:**
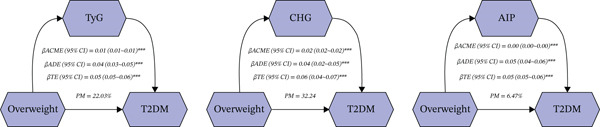
Mediation effects of lipid‐glucose metabolic indices on the association between overweight and T2DM in covariates‐adjusted models. (∗∗∗ for *p* < 0.001).

The mediating roles of the TyG, CHG, and AIP indices in the association between overweight and T2DM were examined using mediation analysis with adjustment for covariates. The results are presented in Figure 3.

All three indices showed statistically significant indirect effects (*p* < 0.001). The CHG index accounted for the largest proportion of the TE of overweight on T2DM (32.24%). The TyG index also exhibited a significant indirect effect, accounting for 22.03% of the TE, whereas the AIP mediated 6.47% of the TE (Figure 3). Complete effect estimates, regression coefficients, and standard errors for all unadjusted and adjusted models are presented in Table S1. The discriminatory ability of the logistic regression models, evaluated by the AUC, are presented in Figure S1.

## 4. Discussion

This study examined the associations of overweight and three lipid‐glucose metabolic indices with T2DM and quantified their mediating roles. The results indicate that overweight was associated with higher odds of T2DM. The TyG index, CHG index, and AIP indices partially mediated this association, with the CHG index showing the largest indirect effect.

### 4.1. Overweight Is Associated With T2DM

Consistent with previous epidemiological evidence [10], overweight was associated with 26% higher odds of T2DM after covariate adjustment. This effect size is statistically significant but modest. This likely reflects the multifactorial nature of T2DM in older adults. RCS results suggested that T2DM risk began to increase when BMI exceeded approximately 24 kg/m^2^. This threshold coincides with the overweight criteria for Asian populations [14]. Visceral adiposity may contribute through chronic inflammation and insulin resistance [27]. Psychosocial factors such as reduced physical activity and dietary challenges may further influence the risk. However, residual confounding by unmeasured comorbidities (cardiovascular, renal disease, etc.) or their treatments cannot be ruled out. These factors may introduce reverse causality or bias [28]. Therefore, longitudinal studies with comprehensive comorbidity data are warranted to clarify these relationships.

### 4.2. TyG Index as a Mediator

We found that overweight was positively associated with the TyG index. Each unit increment in the TyG index was related to 72% higher odds of T2DM. RCS analysis also revealed a nonlinear positive association. T2DM odds rose more steeply above a TyG value of approximately 8.9. Mediation analysis indicated that the TyG index accounted for 22.03% of the TE of overweight on T2DM. This finding suggests that the TyG index may partly capture the metabolic disturbances linking excess adiposity to T2DM. The TyG index is a marker of insulin resistance, it reflects the interplay between adipose tissue dysfunction and systemic glucose metabolism [9]. In the overweight state, hypertrophic adipocytes release could elevate free fatty acids, which can induce insulin resistance in skeletal muscle and liver, promote hepatic gluconeogenesis, and impair glucose uptake. The inclusion of fasting triglycerides in the TyG formula makes it sensitive to this lipid spillover and its glycemic consequences. Although the TyG index has been reported to perform well in predicting T2DM in certain populations [14], its incremental utility beyond conventional risk factors in community‐dwelling older adults remains to be established.

### 4.3. CHG Index as the Strongest Mediator

We found that in the covariate‐adjusted model, each unit increase in the CHG index was associated with a 3.49 times higher odds of T2DM. Visual inspection of the RCS curve suggested that T2DM risk began to increase more steeply once the index exceeded roughly 5.2. Mediation analysis further indicated that the CHG index accounted for 32.24% of the TE of overweight on T2DM. This was the largest PM among the three indices examined. By integrating TC, HDL‐C, and fasting glucose, the CHG index could jointly capture atherogenic dyslipidemia and impaired glucose regulation. These are two processes through which excess adiposity promotes diabetes [29, 30]. In overweight older adults, adipose tissue dysfunction could elevates triglyceride‐rich lipoproteins, lowers HDL‐C, and impairs insulin sensitivity. The CHG index is sensitive to these combined changes. Therefore, it may be a particularly informative marker of the metabolic dysregulation that leads to T2DM [14, 31].

The pattern of a steep increase in odds at CHG values around 5.2 is broadly consistent with prior studies. Zhang et al. [32] reported that T2DM prevalence was significantly elevated at a CHG index > 5.24 in US adults. Li et al. [33] identified a nonlinear turning point for T2DM risk at 5.28 in a nationwide Chinese cohort of middle‐aged and older adults. In addition, Mansoori et al. [11] found that a CHG cutoff of 5.28 demonstrated predictive utility for cardiovascular disease. Our finding extends this literature by quantifying this intermediary pathway in older adults. Given that the CHG index can be readily computed from routine laboratory parameters, it holds potential as a practical tool for metabolic risk stratification. However, the index′s predictive value beyond standard risk factors still requires validation in prospective cohort studies.

### 4.4. AIP as a Significant but Modest Mediator

We also examined the mediating role of the AIP in the association between overweight and T2DM. Overweight was associated with higher AIP levels. Each unit increase in AIP was associated with 16% higher odds of T2DM. Visual inspection of the RCS curve suggested that T2DM risk began to increase more steeply around an AIP of approximately 1.0. Mediation analysis indicated that the AIP accounted for 6.47% of the TE of overweight on T2DM. The AIP is calculated as the log‐transformed ratio of triglycerides to HDL‐C. It reflects atherogenic dyslipidemia characterized by elevated triglycerides, low HDL‐C, and an increase in small, dense LDL‐C particles [34]. This lipid profile is largely driven by insulin resistance. The modest mediation proportion of 6.47% suggests that the specific dyslipidemia captured by the AIP plays an independent but relatively small role in the pathway from overweight to T2DM. It contributes less than the TyG and CHG indices, which reflect broader insulin resistance and combined lipid‐glucose disturbances. The AIP may indicate a more advanced stage of atherogenic dyslipidemia. This stage could impair pancreatic *β*‐cell function and vascular integrity, thereby promoting the development of diabetes [31]. Although its mediating effect was modest, the indirect effect was statistically significant. This supports the notion that, beyond insulin resistance, lipid abnormalities also contribute to the pathogenesis of T2DM.

### 4.5. Limitations and Future Directions

Several limitations should be considered when interpreting our findings. First, the cross‐sectional design of this study precludes causal inference. Although mediation analysis can suggest potential pathways, it cannot establish the temporal sequence among overweight, changes in metabolic indices, and T2DM onset. Second, as noted earlier, data on comorbidities and medication use were unavailable. These factors may influence both body weight and metabolic indices and could introduce residual confounding. Third, the results reflect the conditions among older adults in Shanghai, and may not generalize to other ethnic or geographic groups.

To address these limitations, future research could employ longitudinal designs with repeated assessments. This would capture the dynamic relationships among weight change, metabolic indices, and T2DM development. Incorporating objective measures of body composition beyond BMI, such as dual‐energy X‐ray absorptiometry or magnetic resonance imaging, would provide more precise estimates of adiposity. Comprehensive data on comorbidities and medications should be collected to better control for potential confounding and reverse causality. Finally, validation across diverse populations is also needed to assess the generalizability of our findings. This is particularly important for the prominent mediating role observed for the CHG index.

## 5. Conclusion

In this study, the association between overweight and T2DM was partially mediated by a set of lipid‐glucose metabolic indices. Among them, the CHG index accounted for the largest proportion of the TE, followed by the TyG index and the AIP. This suggests that combined dyslipidemia and impaired glucose regulation, as captured by the CHG index, may represent a particularly important intermediary pathway linking excess adiposity to T2DM. These composite indices each reflect a different facet of metabolic disturbance. They could be considered in future risk assessment frameworks. However, their incremental predictive value beyond conventional risk factors and their clinical utility require validation in prospective, multiethnic cohorts. Such validation is necessary before recommendation for practice can be made.

**Nomenclature**
ACME: average causal mediation effect
ADE: average direct effect
AIP: atherogenic index of plasma
AUC: area under the receiver operating characteristic curve
BMI: body mass index
CHG: cholesterol‐HDL‐glucose
CI: confidence interval
HDL‐C: high‐density lipoprotein cholesterol
ICD‐11: International Classification of Diseases, 11th Revision
LDL‐C: low‐density lipoprotein cholesterol
NCDs: non‐communicable diseases
OR: odds ratio
PM: proportion mediated
RCS: restricted cubic splines
SCDC: Shanghai Municipal Center for Disease Control and Prevention
SD: standard deviation
T2DM: Type 2 diabetes mellitus
TC: total cholesterol
TE: total effect
TG: triglyceride
TyG: triglyceride‐glucose

## Author Contributions

Methodology, Software, Writing‐original draft, Writing‐review and editing, and Visualization: Jia Bo, Hongfei Mo, and Fan Wang. Data analysis: Jia Bo, Hongfei Mo, and Jiayun Zhu. Resources, Data curation, Methodology, and Formal analysis: Fei Wu, Qinghua Yan, Fan Wang, and Minna Cheng. Conceptualization, Supervision, Project administration, and Funding acquisition: Fei Wu, Qinghua Yan, Fan Wang, Minna Cheng and Jia Bo. Jia Bo, Hongfei Mo, and Fei Wu contributed equally to this work.

## Funding

This study was supported by the Noncommunicable Chronic Diseases‐National Science and Technology, 2024ZD0524200; and the 2026 Shanghai University of Medicine & Health Sciences, Faculty Talent Hundred Talents Pool Program, A3‐2601‐26‐311009.

## Ethics Statement

This study has been approved by the Ethics Committee of SCDC (approval number: KY‐2024‐52‐C). This study was strictly performed according to the Declaration of Helsinki. All participants provided written informed consent before participating.

## Consent

The authors have nothing to report.

## Conflicts of Interest

The authors declared no conflicts of interest.

## Supporting Information

Additional supporting information can be found online in the Supporting Information section.

## Supporting information


**Supporting Information 1** Table S1: Mediation roles of TyG, CHG, and AIP in the association of overweight and T2DM. (Word file).

**Supporting Information 2** Figure S1: ROC curves AUC for the logistic regression models evaluating the associations of (A) TyG, (B) CHG, and (C) AIP with T2DM. (Word file).

## Data Availability

The data that support the findings of this study are available from the corresponding author upon reasonable request.
