# Supplementary material for: Mediation by Lipid‐Glucose Metabolic Indices in the Association Between Overweight and T2DM Among Shanghai Community‐Dwelling Older Adults
Source: J Diabetes Res. 2026 Jun 26;2026:8449810. doi: 10.1155/jdr/8449810 (PMC13307189; doi:10.1155/jdr/8449810)
Supplement: Supplementary file 1 — Supporting Information 1 Table S1: Mediation roles of TyG, CHG, and AIP in the association of overweight and T2DM. (Word file). [file JDR-2026-8449810-s002.docx]

| **Supplementary Table S1:** Mediation roles of TyG, CHG, and AIP in the association of overweight and T2DM | | | | | | | |
| --- | --- | --- | --- | --- | --- | --- | --- |
|  | ACME | | ADE | | TE | | PM |
|  | Estimate  (95% CI) | *P* | Estimate  (95% CI) | *P* | Estimate  (95% CI) | *P* | Estimate proportion |
| TyG | 0.01  (0.01, 0.01) | <0.001 | 0.04  (0.03, 0.05) | <0.001 | 0.05  (0.05, 0.06) | <0.001 | 22.03% |
| CHG | 0.02  (0.02, 0.02) | <0.001 | 0.04  (0.03, 0.05) | <0.001 | 0.06  (0.04, 0.07) | <0.001 | 32.24% |
| AIP | 0.00  (0.00, 0.00) | <0.001 | 0.05  (0.04, 0.06) | <0.001 | 0.05  (0.05, 0.06) | <0.001 | 6.47% |
| Adjusted for covariates: age, sex, marital status, education, smoking, alcohol consumption, physical activity.  ACME: average causal mediation effect, ADE: average direct effect, TE: total effect, PM: proportion mediated, T2DM: type 2 diabetes mellitus, TyG: triglyceride-glucose index, CHG: cholesterol-HDL-glucose index, AIP: atherogenic index of plasma. | | | | | | | |
