# Supplementary material for: Mediation by Lipid‐Glucose Metabolic Indices in the Association Between Overweight and T2DM Among Shanghai Community‐Dwelling Older Adults
Source: J Diabetes Res. 2026 Jun 26;2026:8449810. doi: 10.1155/jdr/8449810 (PMC13307189; doi:10.1155/jdr/8449810)
Supplement: Supplementary file 2 — Supporting Information 2 Figure S1: ROC curves AUC for the logistic regression models evaluating the associations of (A) TyG, (B) CHG, and (C) AIP with T2DM. (Word file). [file JDR-2026-8449810-s001.docx]

| A | B | C |
| --- | --- | --- |
| 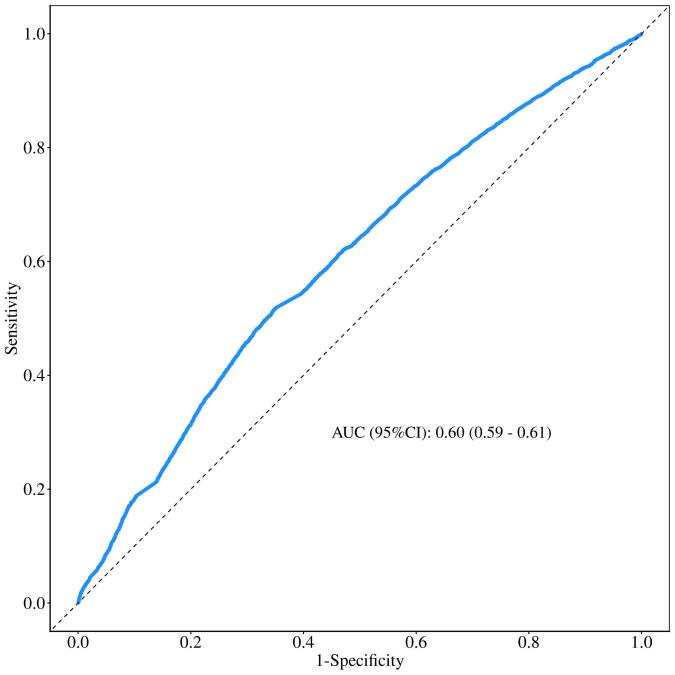 | 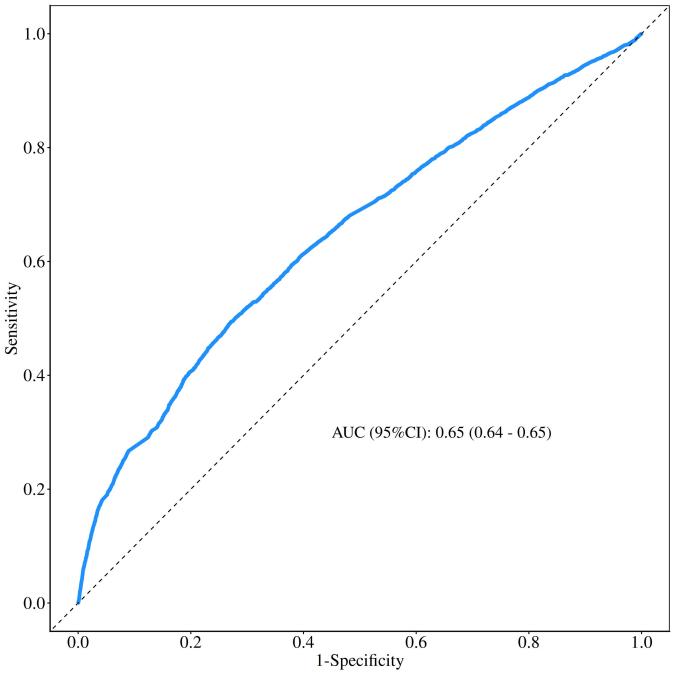 | 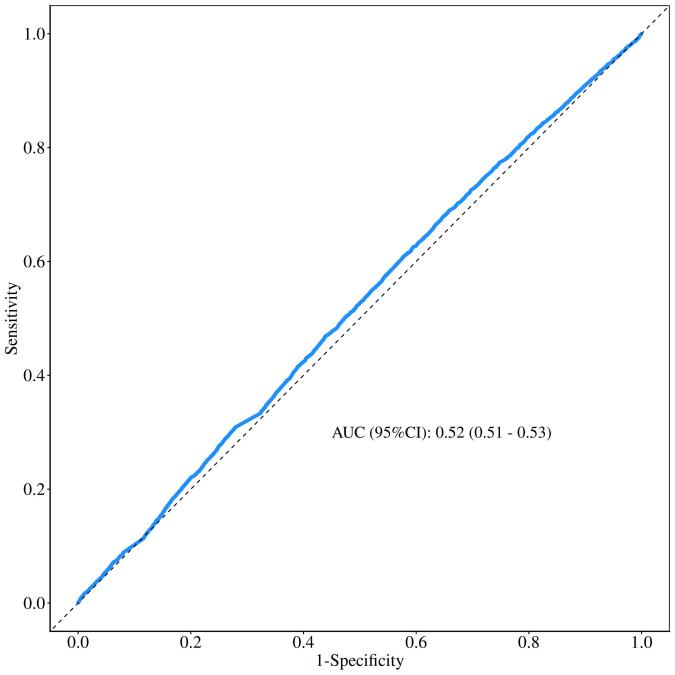 |
| **Supplementary Figure S1:** ROC curves AUC for the logistic regression models evaluating the associations of TyG (A), CHG (B), and AIP (C) with T2DM. | | |
